# Supplementary material for: Identification of Nanoparticle Properties for Optimal Drug Delivery across a Physiological Cell Barrier
Source: Pharmaceutics. 2023 Jan 6;15(1):200. doi: 10.3390/pharmaceutics15010200 (PMC9865979; doi:10.3390/pharmaceutics15010200)
Supplement: Supplementary file 1 [file pharmaceutics-15-00200-s001.zip › File S1 - Nanoparticle Synthesis.pdf]

pharmaceutics

Article

Identification of Nanoparticle Properties for Optimal Drug Delivery Across a Physiological Cell Barrier

Aisling M. Ross[1,2,*], Rachel M. Cahalane[1,2,3], Darragh R. Walsh[1,2], Andreas M. Grabrucker[1,4,5], Lynnette Marcar[6] and John J. E. Mulvihill[1,2,4,*]

File S1 – Nanoparticle Synthesis

Gold (Au) Nanoparticles

Gold nanoparticles (NPs) were purchased from Applied Nanoparticles (Barcelona, Spain). The NPs were citrate stabilized, supplied in 2.2 mM sodium citrate solution at concentrations of 1.23 mg/mL (50 nm), 0.89 mg/mL (20 nm), and 0.73 mg/mL (5 nm).

Silver (Ag) Nanoparticles

20 and 50 nm silver nanoparticles were purchased from Applied Nanoparticles (Barcelona, Spain) and 5 nm silver nanoparticles (SEPE5-25M-NCX) were purchased from nanoComposix (San Diego, CA, USA). PVP stabilized nanoparticles were supplied at concentrations of 1.1 mg/mL (50 nm) and 1.065 mg/mL (20 nm) in 5 mM sodium citrate and 4.92 mg/mL (5 nm) in water.

Zinc Oxide (ZnO) Nanoparticles

50 nm

100 mL of a 0.1 M solution of zinc chloride (Sigma Aldrich Z0152, 97% purity) and 100 mL of a 0.2 M solution of NaOH (Sigma Aldrich 221465, >97% purity) were prepared in dH_2O. The NaOH solution was added at a rate of 10 mL/min to the $ZnCl_2$ solution with constant stirring at a rate of 300 rpm. The solution was heated to 60 ⁰C and maintained for 2 hours. The reaction was stopped by transferring the reaction flask to an ice bath. The particles were collected by centrifugation for 10 mins at 100 xg and washed 3 times with dH_2O to remove residual precursor. The particles were then dried overnight in an incubator at 60 ⁰C and calcined in a furnace (OTF-1200X, MTI Corporation, USA) at 450 ⁰C for 2 h to convert the sample completely from $Zn(OH)_2$ to ZnO.

20 nm

1.095 g of zinc chloride was dissolved in 75 mL of methanol (Fisher Scientific 10675112, >95% purity), and 0.774 g of KOH (Fisher Scientific 10509390) was dissolved in 39 mL of methanol. Both solutions were stirred at 50-55 ⁰C for 30 mins. The KOH was added to the $ZnCl_2$ solution at a rate of 10 mL/min with constant stirring at a rate of 500 rpm. The solution was maintained between 55-65 ⁰C for 2 h and then transferred to an ice bath. The particles were collected by centrifugation for 10 mins at 1000 xg and washed 3 times with methanol to remove residual precursor. The particles were dried overnight in a Memmert oven (Memmert GmbH, Germany) at 80 ⁰C and then calcined at 300 ⁰C for 3 h to convert the sample completely from $Zn(OH)_2$ to ZnO.

5 nm

1.095 g of zinc chloride (Fisher Scientific 11407737) was dissolved in 75 mL of methanol and 0.774 g of KOH was dissolved in 39 mL of methanol. Both solutions were stirred at 55-60 ⁰C for 30 min. The KOH was added to the $ZnCl_2$ solution at a rate of 10 mL/min

with constant stirring at a rate of 500 rpm. Following addition of the KOH, the reaction was maintained at 55-60 ^{0}C for a further 3 min and then transferred to an ice bath. The particles were collected by centrifugation for 10 min at 2000 xg and washed 3 times with methanol to remove residual precursor. The particles were dried overnight in a Memmert oven at 80 ^{0}C and then calcined at 300 ^{0}C for 3 h to convert the sample completely from $Zn(OH)_2$ to ZnO.

Iron Oxide (Fe_2O_3) Nanoparticles

50 nm

97.35 µL of ammonium hydroxide was added to 5 mL dH_2O and mixed for 30 min. 0.13515 g of $FeCl_3.6H_2O$ was dissolved in 5 mL of dH_2O with stirring for 30 min. The $FeCl_3.6H_2O$ solution was then added to the ammonium hydrate solution and stirred for a further 30 min before heating for 24 h in a Teflon lined autoclave at 120 ^{0}C. The particles were dried overnight and collected.

250 mg of sample was ball milled at 25 Hz for 10 min in a 25 mL stainless steel jar using a Retsch Oscillating Mill MM400 (RETSCH GmbH, Germany). The sample was suspended in 10 mL of sterile dH_2O and sonicated for 1 h in a Branson 3800 sonicating bath. The sample was then centrifuged at 50 xg to remove the larger particles. The supernatant was collected and centrifuged at 100 xg for 10 min and the pellet was dried at room temperature overnight.

20 nm

A solution of 0.1 M $FeCl_3$ (Sigma Aldrich 8039451000) and 0.2 M $FeCl_2.4H_2O$ (Fisher Scientific 11497797) was prepared by dissolving 0.4055 g of $FeCl_3$ and 0.994 g $FeCl_2.4H_2O$ in 25mL of dH_2O. 25 mL of a 0.2 M NaOH solution was added to the iron chloride solution at a rate of 5 mL/min at 50-60 ^{0}C with constant stirring at 500 rpm. The reaction was maintained between 50-60 ^{0}C with constant stirring for 2 h before transferring to an ice bath. The particles were then centrifuged at 1000 xg for 10 min to remove residual precursor and washed 3 times with dH_2O. Particles were dried overnight at 80 ^{0}C and calcined at 300 ^{0}C for 3 h.

5 nm

A 25 mL solution of 0.1 M $FeCl_3$ sand 0.2 M $FeCl_2.4H_2O$ was prepared as before. 25 mL of a 0.2 M NaOH solution was added to the iron chloride solution at a rate of 5 mL/min at 50-60 ^{0}C with constant stirring at 500 rpm. The reaction was stopped immediately by transferring to an ice bath. The particles were then centrifuged at 1000 xg for 10 min to remove residual precursor and washed 3 times with dH_2O. Particles were then dried overnight at 80 ^{0}C and calcined at 300 ^{0}C for 3 h.

Titanium Dioxide (TiO_2) Nanoparticles

5 mL of titanium tetraisopropoxide (TTIP) (Sigma Aldrich 205273) was added to 15 mL of isopropanol (Fisher Scientific 10588630) and mixed on a magnetic stirrer until homogeneous. Ammonium hydroxide (Sigma Aldrich 221228) was added to dH_2O until it reached pH 10. The TTIP solution was added to the basic water with vigorous stirring. The solution was heated to 70 ^{0}C and maintained for 18 h. The reaction was quenched by transferring to an ice bath. The particles were collected by centrifugation at 2000 xg for 10 min and washed 3 times in ethanol. The particles were dried overnight in a vacuum oven (YHCHM Model:DZF-6050) at 35 ^{0}C. The particles were then calcined for 2 h at 100 ^{0}C (5 nm), 650 ^{0}C (20 nm) and 850 ^{0}C (50 nm).
