# Supplementary material for: Identification of Nanoparticle Properties for Optimal Drug Delivery across a Physiological Cell Barrier
Source: Pharmaceutics. 2023 Jan 6;15(1):200. doi: 10.3390/pharmaceutics15010200 (PMC9865979; doi:10.3390/pharmaceutics15010200)
Supplement: Supplementary file 1 [file pharmaceutics-15-00200-s001.zip › File S2 - Nanoparticle Characterisation.pdf]

pharmaceutics

Article

Identification of Nanoparticle Properties for Optimal Drug Delivery Across a Physiological Cell Barrier

Aisling M. Ross[1,2,*], Rachel M. Cahalane[1,2,3], Darragh R. Walsh[1,2], Andreas M. Grabrucker[1,4,5], Lynnette Marcar[6] and John J. E. Mulvihill[1,2,4,*]

File S2 – Nanoparticle Characterisation

Nanoparticle Sterilisation

All NPs were sterilised before use in all experiments. The silver, gold, and iron oxide NPs were all tyndallized. Aliquots of the gold and silver NPs were taken from the stock suspension purchased. Iron oxide NPs were suspended in dH_2O at a concentration of 1 mg/mL. For sterilization, the NP solutions were placed in a water bath at 100 ^{0}C for 30 min. The suspension was then transferred to an incubator at 37 ^{0}C for 24 h. This cycle was repeated 3 times over the course of 3 consecutive days. In this process the heating cycles kill any live pathogens. Then during the incubation phase, any potential spores are encouraged to germinate and then killed in the subsequent heating cycle.

Zinc oxide and titanium dioxide NPs could not be sterilized by tyndallization as these NPs were not stable for long periods in water. Instead, the dried NP powders were weighed out and placed in Eppendorf tubes which were then sterilised by UV radiation for 30 min in a Level 2 Biosafety Cabinet.

Inductively Coupled Plasma-Mass Spectroscopy

For ICP-MS analysis, the digested sample is introduced to the instrument via a peristaltic pump where it mixes with an internal standard. The mixture is then nebulised and passed through a cooled spray chamber to remove large droplets. The remainder of the aerosol is transported to a torch. In the torch, argon gas is subjected to a magnetic field, generated by a radio-frequency coil. This causes the gas to form a stable plasma. The high temperature plasma causes the sample aerosol to ionise. The plasma and aerosol are then transported via ion optics to a quadrupole to determine their mass:charge ratio. This is used to determine the identity of each element in the sample. The samples were quantified for gold (Au NPs), silver (Ag NPs), iron (Fe_2O_3 NPs), titanium (TiO_2 NPs), and zinc (ZnO NPs). The average permeation was then expressed as a percentage of the original loaded quantity. Table S1 summarises the limits of quantification for the ICP-MS analysis of the nanoparticles.

Table S1. The lower and upper limits of quantification for inductively-coupled plasma mass spectrometry of the nanoparticles tested. Note: Iron oxide, titanium dioxide and zinc oxide nanoparticles were quantified by analysing the amount of iron, titanium and zinc, respectively.

Sample	Lower Limit of Quantification (ng/mL)	Upper Limit of Quantification (ng/mL)
Gold	2.5	250
Silver	2.5	250
Iron	100	10,000
Titanium	100	10,000
Zinc	25	375
