# Supplementary material for: Identification of Nanoparticle Properties for Optimal Drug Delivery across a Physiological Cell Barrier
Source: Pharmaceutics. 2023 Jan 6;15(1):200. doi: 10.3390/pharmaceutics15010200 (PMC9865979; doi:10.3390/pharmaceutics15010200)
Supplement: Supplementary file 1 [file pharmaceutics-15-00200-s001.zip › File S3 ¿C Nanoparticle Permeation and Resistance.pdf]

pharmaceutics

MDPI

Article

Identification of Nanoparticle Properties for Optimal Drug Delivery Across a Physiological Cell Barrier

Aisling M. Ross[1,2,*], Rachel M. Cahalane[1,2,3], Darragh R. Walsh[1,2], Andreas M. Grabrucker[1,4,5], Lynnette Marcar[6] and John J. E. Mulvihill[1,2,4,*]

File S3 – Nanoparticle Permeation and Resistance

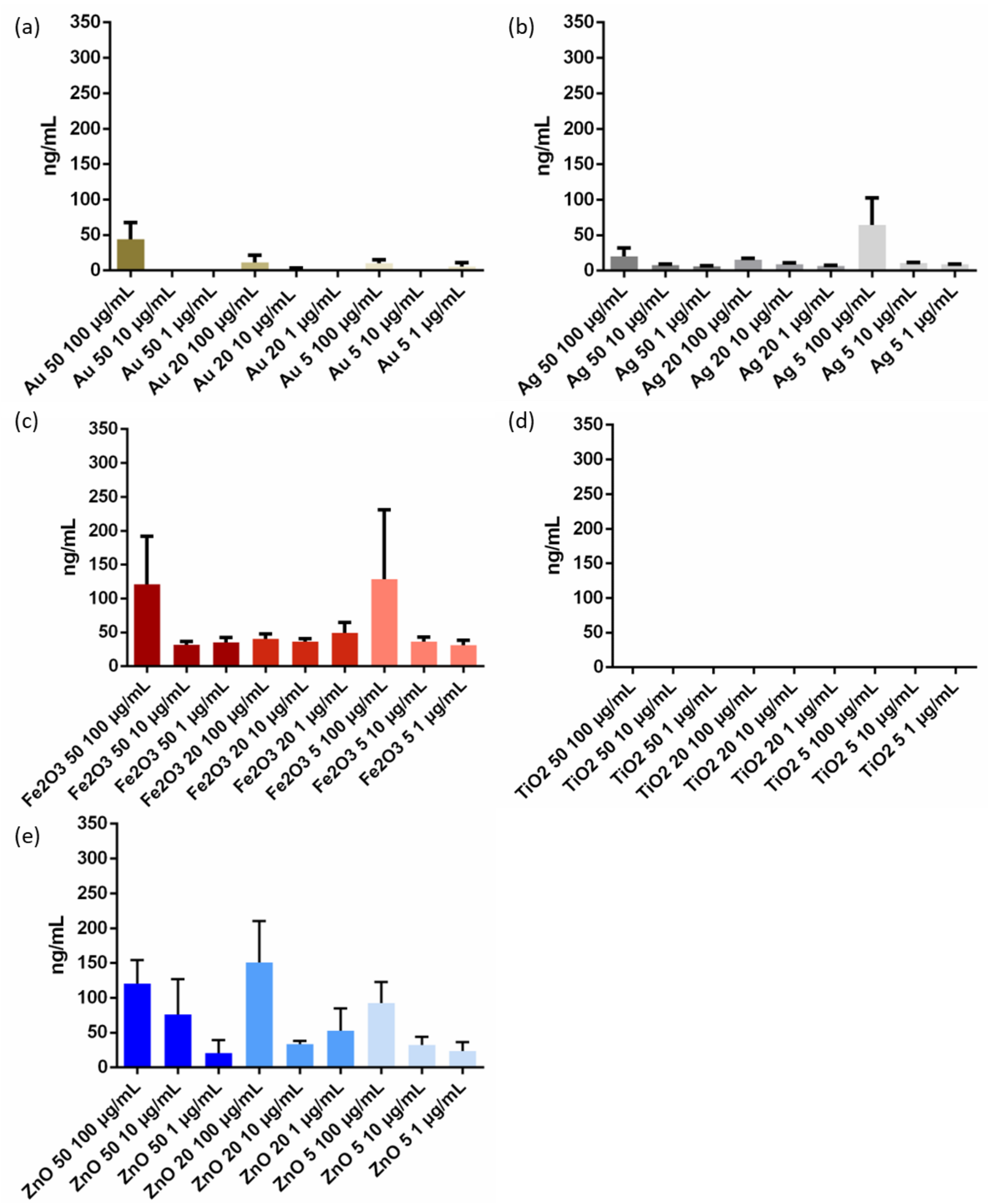

Figure S1. Quantity of nanoparticle permeation across the cell barrier following 12 hour incubation with (a) gold, (b) silver, (c) iron (III) oxide, (d) titanium dioxide, (e) zinc oxide nanoparticles. Colour Key for all graphs: Au = Gold, Ag = Grey, Fe_2O_3 = Red, TiO_2 = Green, and ZnO = Blue.

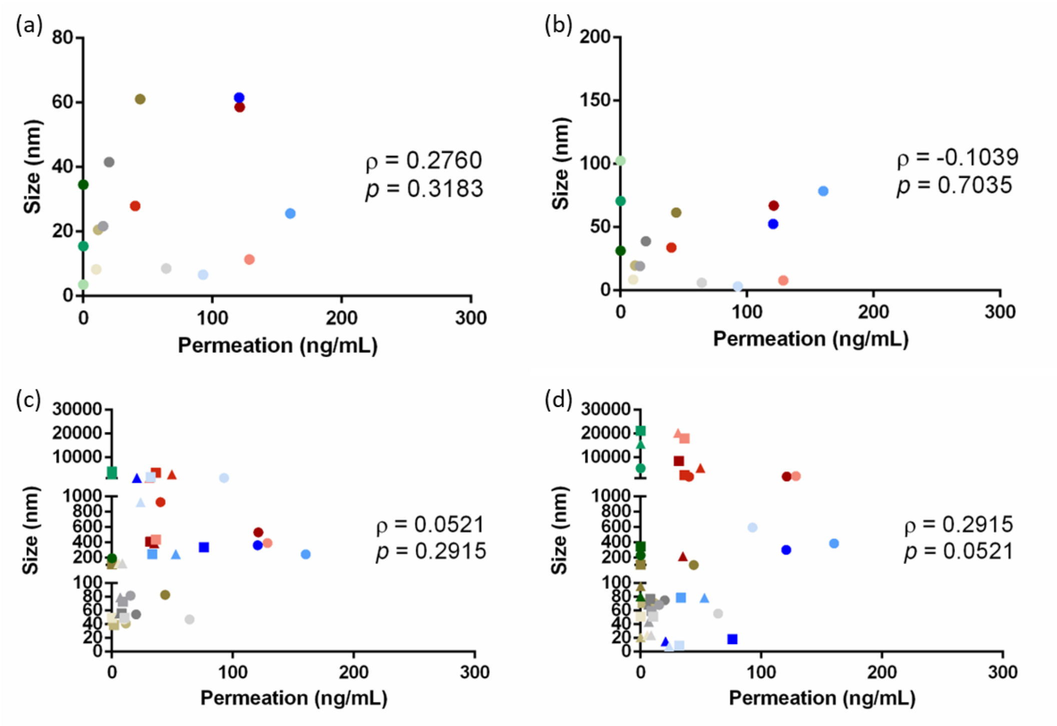

Figure S2. Correlations between nanoparticle (NP) permeability and NP properties. (a) Spearman's correlation between NP permeability and NP size (measured via TEM) in water. (b) Spearman's correlation between NP permeability and NP size (measured via TEM) in media. (c) Spearman's correlation between NP permeability and NP size (measured via DLS) in water. (d) Spearman's correlation between NP permeability and NP size (measured via DLS) in media. ● = 100 µg/mL NP dispersion, ■ = 10 µg/mL NP dispersion, Δ = 1 µg/mL NP dispersion, TEM = Transmission electron microscopy, DLS = Dynamic light scattering. Colour Key for all graphs: Au = Gold, Ag = Grey, Fe_2O_3 = Red, TiO_2 = Green, and ZnO = Blue.

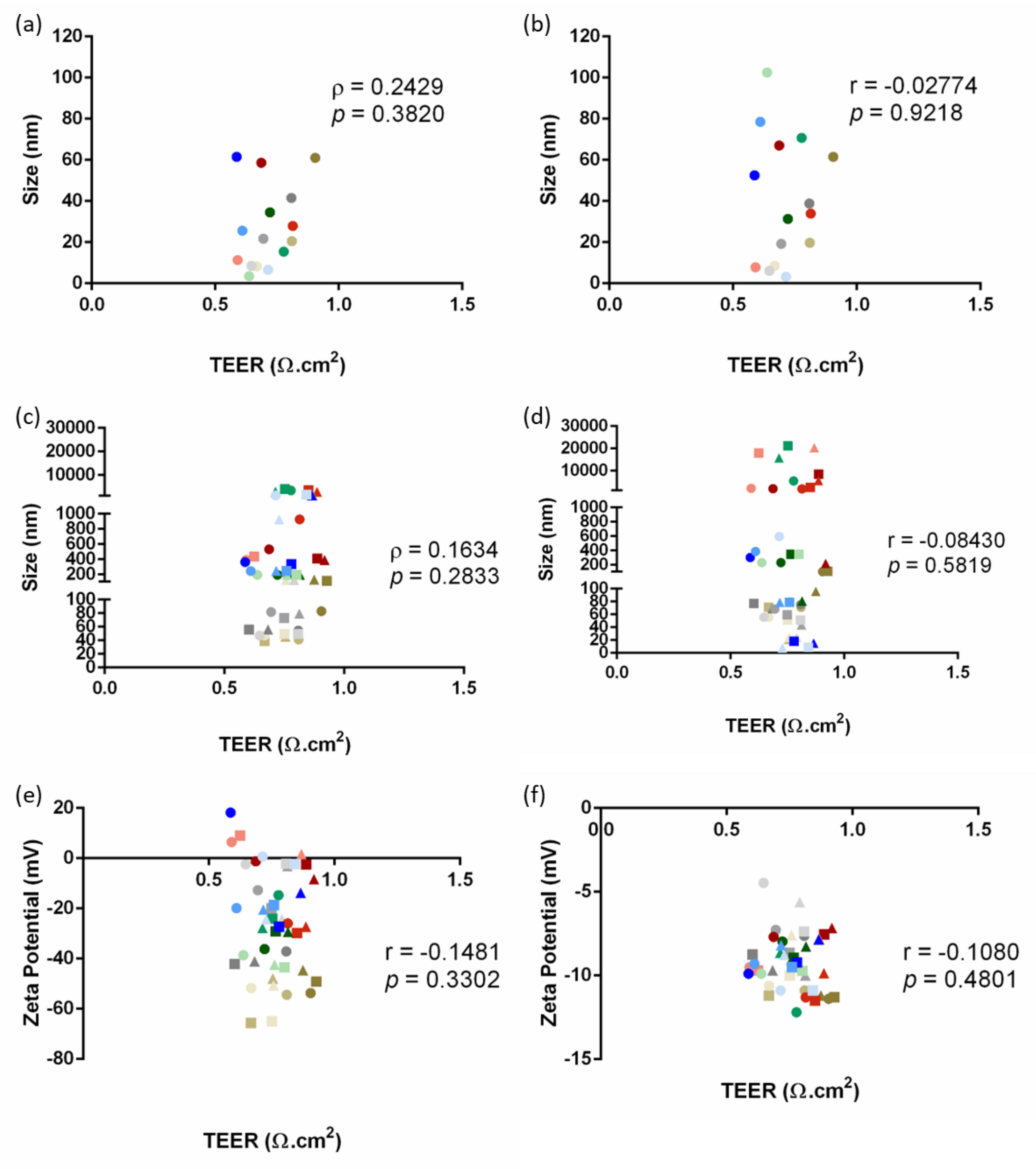

Figure S3. Correlations between changes to transendothelial electrical resistance (TEER) and nanoparticle (NP) properties. (a) Spearman's correlation between TEER and NP size (measured via TEM) in water. (b) Pearson's correlation between TEER and NP size (measured via TEM) in media. (c) Spearman's correlation between TEER and NP size (measured via DLS) in water. (d) Pearson's correlation between TEER and NP size (measured via DLS) in media. (e) Pearson's correlation between TEER and NP zeta potential in water. (f) Pearson's correlation between TEER and NP zeta potential in media. ● = 100 µg/mL NP dispersion, ■ = 10 µg/mL NP dispersion, Δ = 1 µg/mL NP dispersion, TEM = Transmission electron microscopy, DLS = Dynamic light scattering. Colour Key for all graphs: Au = Gold, Ag = Grey, Fe_2O_3 = Red, TiO_2 = Green, and ZnO = Blue.
