# Supplementary material for: Identification of Nanoparticle Properties for Optimal Drug Delivery across a Physiological Cell Barrier
Source: Pharmaceutics. 2023 Jan 6;15(1):200. doi: 10.3390/pharmaceutics15010200 (PMC9865979; doi:10.3390/pharmaceutics15010200)
Supplement: Supplementary file 1 [file pharmaceutics-15-00200-s001.zip › File S4 ¿C Cell Viability.pdf]

pharmaceutics

MDPI

Article

Identification of Nanoparticle Properties for Optimal Drug Delivery Across a Physiological Cell Barrier

Aisling M. Ross[1,2,*], Rachel M. Cahalane[1,2,3], Darragh R. Walsh[1,2], Andreas M. Grabrucker[1,4,5], Lynnette Marcar[6] and John J. E. Mulvihill[1,2,4,*]

File S4 – Cell Viability

The viability of the various cells were evaluated and statistically analysed against different NP parameters (size, zeta potential).

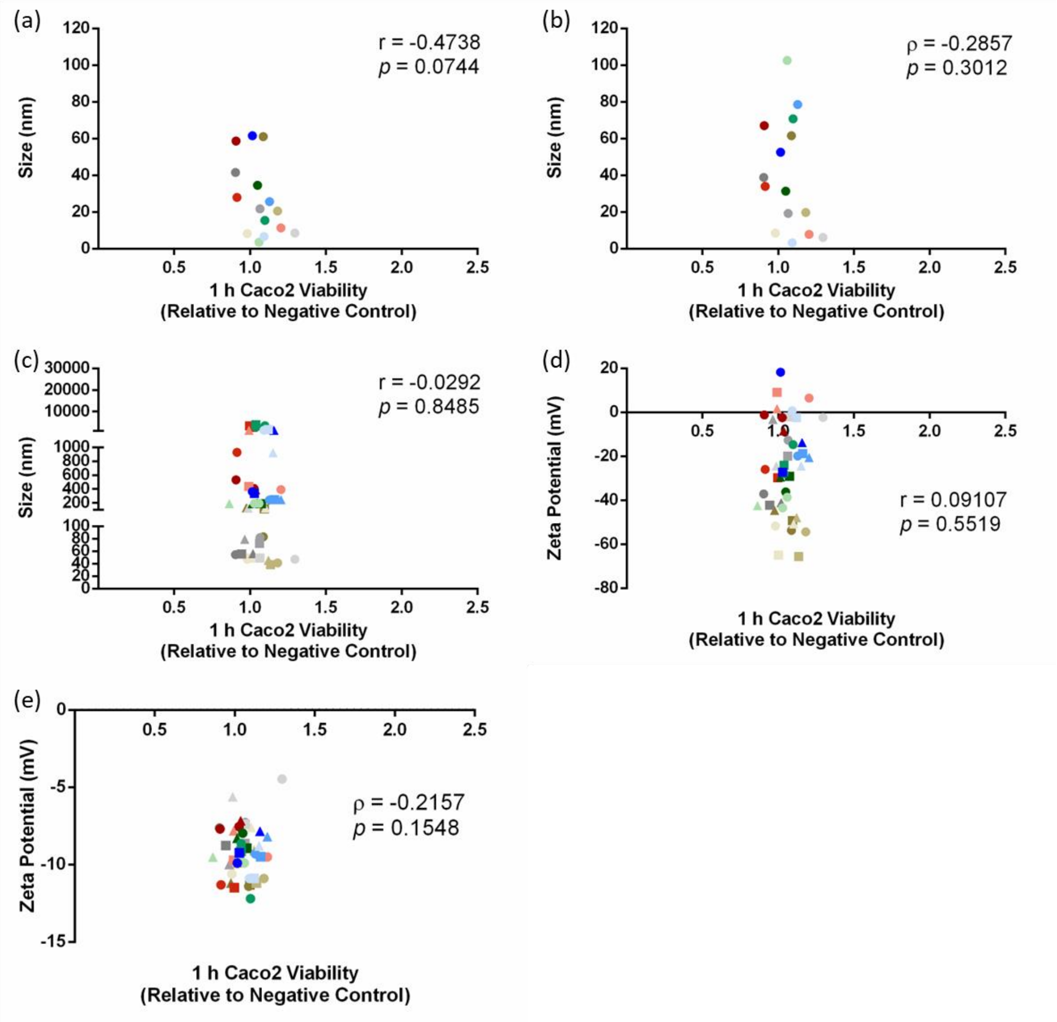

Figure S4. Correlations between changes to Caco-2 cell viability after 1 h nanoparticle (NP) treatment and NP properties. (a) Pearson's correlation between Caco-2 cell viability after 1 h treatment and NP size (measured via TEM) in water. (b) Spearman's correlation between Caco-2 cell viability after 1 h treatment and NP size (measured via TEM) in media. (c) Pearson's correlation between Caco-2 cell viability after 1 h treatment and nanoparticle size (measured via DLS) in water. (d) Pearson's correlation between Caco-2 cell viability after 1 h treatment and nanoparticle zeta potential measured in water. (e) Spearman's correlation between Caco-2 cell viability after 1 h treatment and nanoparticle zeta potential measured in media. ● = 100 µg/mL NP dispersion, ■ = 10 µg/mL NP dispersion, Δ = 1 µg/mL NP dispersion, TEM = Transmission electron microscopy, DLS = Dynamic

light scattering. Colour Key for all graphs: Au = Gold, Ag = Grey, Fe_2O_3 = Red, TiO_2 = Green, and ZnO = Blue.

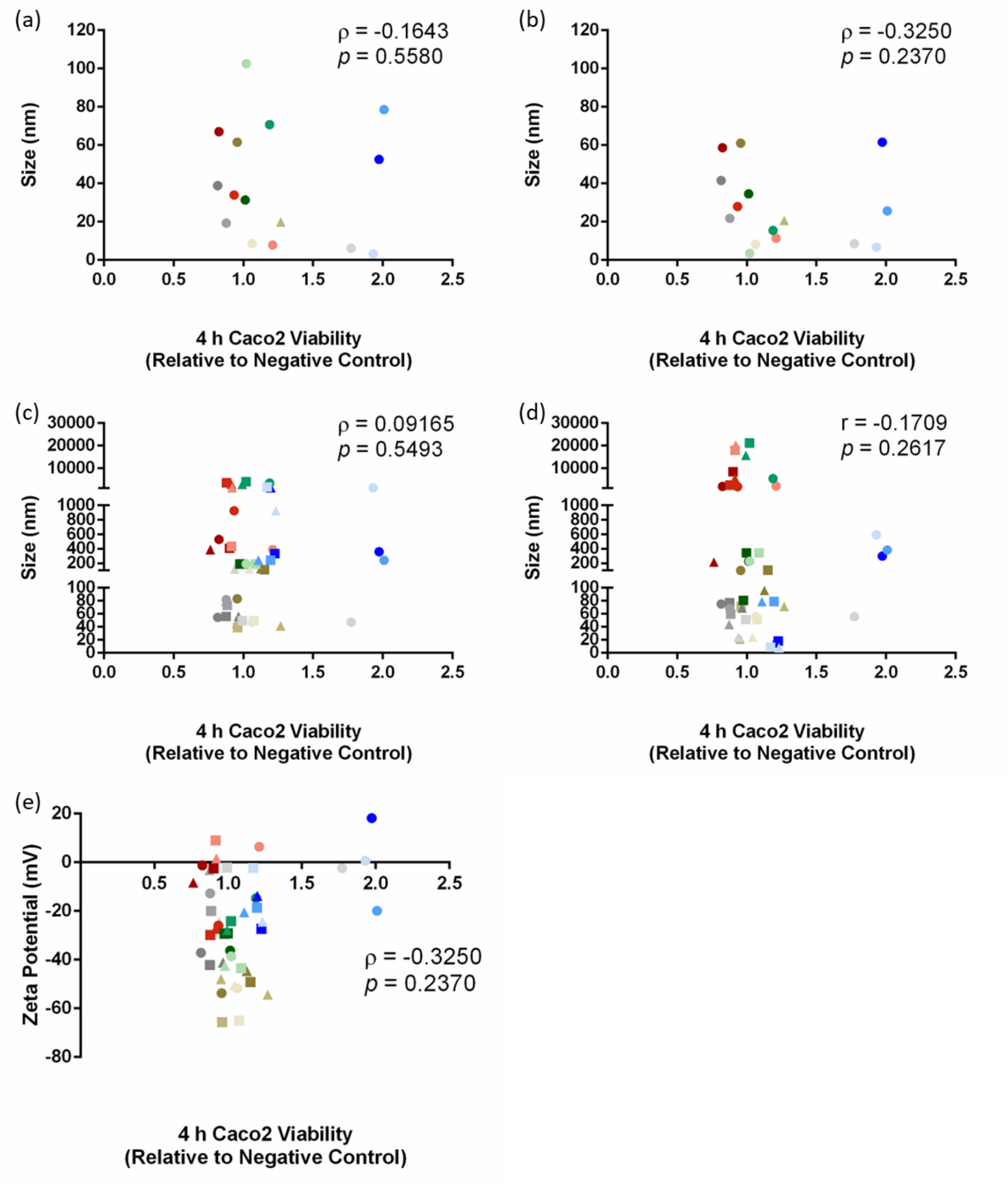

Figure S5. Correlations between changes to Caco-2 cell viability after 4 h nanoparticle (NP) treatment and NP properties. (a) Spearman's correlation between Caco-2 cell viability after 4 h treatment and NP size (measured via TEM) in water. (b) Spearman's correlation between Caco-2 cell viability after 4 h treatment and NP size (measured via TEM) in media. (c) Spearman's correlation between Caco-2 cell viability after 4 h treatment and NP size (measured via DLS) in water. (d) Pearson's correlation between Caco-2 cell viability after 4 h treatment and NP size (measured via DLS) in media. (e) Spearman's correlation between Caco-2 cell viability after 4 h treatment and NP zeta potential in water. ● = 100 μg/mL NP dispersion, ■ = 10 μg/mL NP dispersion, Δ = 1 μg/mL NP dispersion, TEM = Transmission electron microscopy, DLS = Dynamic light scattering. Colour Key for all graphs: Au = Gold, Ag = Grey, Fe_2O_3 = Red, TiO_2 = Green, and ZnO = Blue.

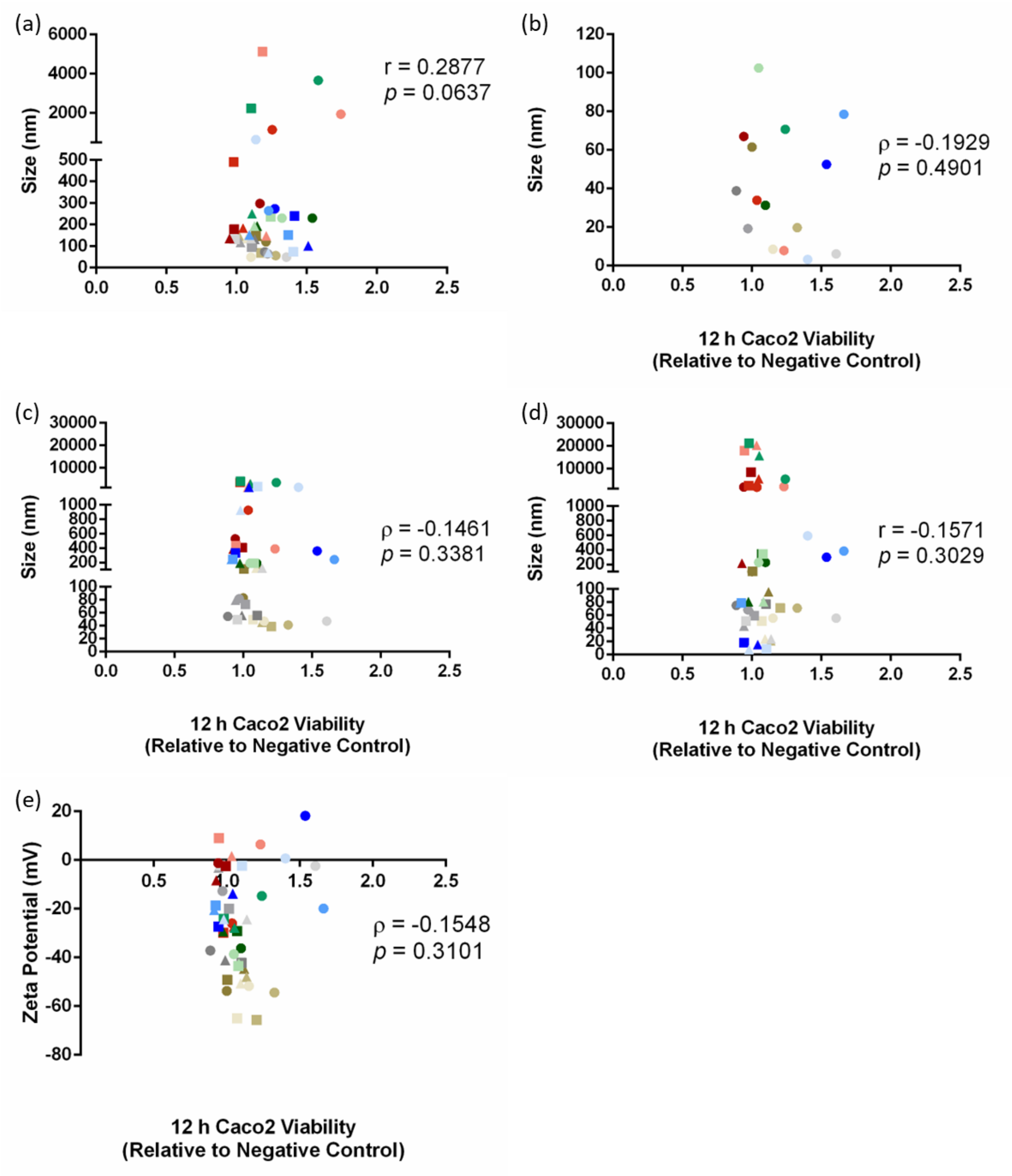

Figure S6. Correlations between changes to Caco-2 cell viability after 12 h nanoparticle (NP) treatment and NP properties. (a) Pearson's correlation between Caco-2 cell viability after 12 h treatment and NP size (measured via TEM) in water. (b) Spearman's correlation between Caco-2 cell viability after 12 h treatment and NP size (measured via TEM) in media. (c) Spearman's correlation between Caco-2 cell viability after 12 h treatment and NP size (measured via DLS) in water. (d) Pearson's correlation between Caco-2 cell viability after 12 h treatment and NP size (measured via DLS) in media. (e) Spearman's correlation between Caco-2 cell viability after 12 h treatment and NP zeta potential in water. ● = 100 µg/mL dose NP dispersion, ■ = 10 µg/mL NP dispersion, Δ = 1 µg/mL NP dispersion, TEM = Transmission electron microscopy, DLS = Dynamic light scattering. Colour Key for all graphs: Au = Gold, Ag = Grey, Fe_2O_3 = Red, TiO_2 = Green, and ZnO = Blue.

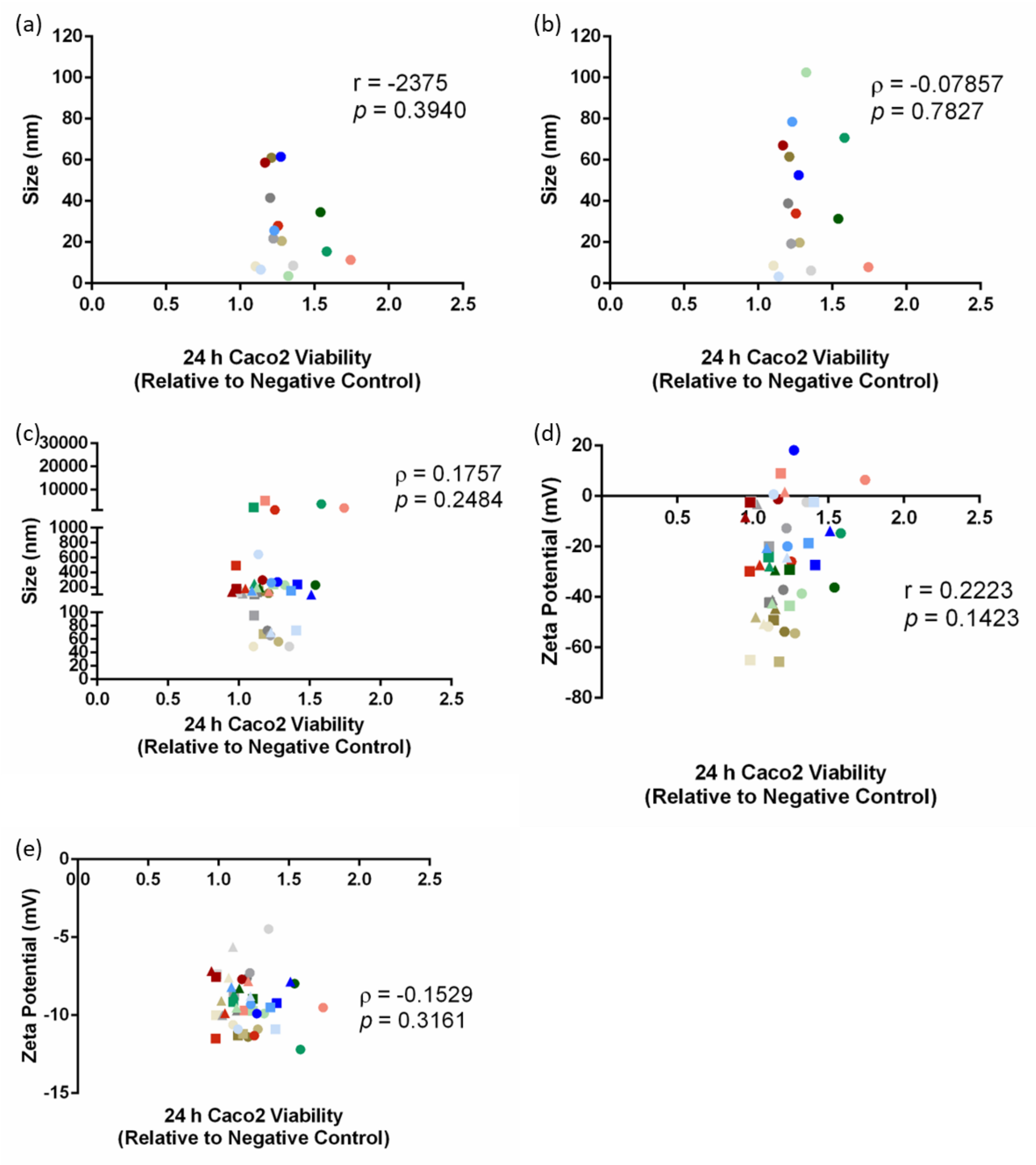

Figure S7. Correlations between changes to Caco-2 cell viability after 24 h nanoparticle (NP) treatment and NP properties. (a) Pearson's correlation between Caco-2 cell viability after 24 h treatment and NP size (measured via TEM) in water. (b) Spearman's correlation between Caco-2 cell viability after 24 h treatment and NP size (measured via TEM) in media. (c) Spearman's correlation between Caco-2 cell viability after 24 h treatment and NP size (measured via DLS) in water. (d) Pearson's correlation between Caco-2 cell viability after 24 h treatment and NP zeta potential measured in water. (e) Spearman's correlation between Caco-2 cell viability after 24 h treatment and NP zeta potential measured in media. ● = 100 µg/mL NP dispersion, ■ = 10 µg/mL NP dispersion, Δ = 1 µg/mL NP dispersion, TEM = Transmission electron microscopy, DLS = Dynamic light scattering. Colour Key for all graphs: Au = Gold, Ag = Grey, Fe_2O_3 = Red, TiO_2 = Green, and ZnO = Blue.
